# Does Workplace Social Capital Associate with Hazardous Drinking Among Chinese Rural-Urban Migrant Workers?

**DOI:** 10.1371/journal.pone.0115286

**Published:** 2014-12-12

**Authors:** Junling Gao, Scott R. Weaver, Hua Fua, Zhigang Pan

**Affiliations:** 1 School of Public Health, Fudan University, Key Laboratory of Public Health Safety, Ministry of Education, Shanghai, China; 2 School of Public Health, Georgia State University, Atlanta, Georgia, United States of America; 3 Department of General Medicine, Affiliated Zhongshan Hospital of Fudan University, Shanghai, China; University of South Carolina, United States of America

## Abstract

**Background:**

The present study sought to investigate the associations between workplace social capital and hazardous drinking (HD) among Chinese rural-urban migrant workers (RUMW).

**Methods:**

A cross sectional study with a multi-stage stratified sampling procedure was conducted in Shanghai during July 2012 to January 2013. In total, 5,318 RUMWs from 77 workplaces were involved. Work-place social capital was assessed using a validated and psychometrically tested eight-item measure. The Chinese version of Alcohol Use Disorders Identification Test (AUDIT) was used to assess hazardous drinking. Control variables included gender, age, marital status, education level, salary, and current smoking. Multilevel logistic regression analysis was conducted to test whether individual- and workplace-level social capital was associated with hazardous drinking.

**Results:**

Overall, the prevalence of HD was 10.6%. After controlling for individual-level socio-demographic and lifestyle variables, compared to workers in the highest quartile of individual-level social capital, the odds of HD for workers in the three bottom quartiles were 1.13(95%CI: 1.04–1.23), 1.17(95%CI: 1.05–1.56) and 1.26(95%CI: 1.13–1.72), respectively. However, contrary to hypothesis, there was no relationship between workplace-level social capital and hazardous drinking.

**Conclusions:**

Higher individual-level social capital may protect against HD among Chinese RUMWs. Interventions to build individual social capital among RUMWs in China may help reduce HD among this population.

## Introduction

Excess alcohol consumption is the world's third largest risk factor for disease and disability; almost 4% of all deaths worldwide are attributed to alcohol [1]. In 2002, the data of China National Nutrition and Health Survey indicated that the prevalence of drinking among adults aged 15 years and older was 21.0% [2], and drinking was second leading risk factor of global burden of disease [3]. Hazardous drinking (HD) is a pattern of alcohol consumption that increases the risk of harmful consequences, including violence, child neglect and abuse, and absenteeism in the workplace for the user or others [1], [4]. Because HD are more common than alcohol dependence, and may be more responsive to intervention [5], so HD is of public health significance despite the absence of any current disorder in the individual user.

Massive rural-urban migration has been stimulated by the rapid modernization and industrialization that is transforming China. It was estimated that there were 160 million migrants who working in urban area, which might represent approximately 25% of the Chinese working population in 2010 [6]. Those rural-urban migrants form a special and vulnerable population group called rural-urban migrant workers (RUMW), who move from rural to urban areas in search of employment and higher living standards without first establishing permanent urban residence [7]. Compared with urban residents, rural-urban migrants are more vulnerable to HD because of the greater social, economic, and work related stressors they experience [8]. Previous studies indicated that 27.0% of rural-urban migrants were intoxicated at least once every month [9] and 57% of migrant women in entertainment venues were hazardous drinkers [10]. In recent years, researchers of public health are paying much attention to social capital, and consider social capital is one social determinant of health and health related behaviors [11], [12]. Previous Studies also demonstrated social support, as a mechanism linking social capital and health, was negatively associated to HD [13]–[15]. Some studies conducted in rural China have generally found a positive association between social capital and positive health [16]–[18]. A study of Chinese offshore oil workers similarly found that current drinking was negatively related to emotional support from friends [19]. Thus, the emerging literature suggests social capital may convey protective health benefits, including lower risk for hazardous dinking.

Social capital is defined as those features of social structures, such as levels of interpersonal trust and norms of reciprocity and mutual aid, which constitute resources for individuals and facilitate collective action [20]–[22]. Social capital can be divided into structural and cognitive components. The structural component includes social interaction in networks giving access to resources. The values, norms and reciprocity, regarded as the cognitive component of social capital, can be seen as a resource held between individuals interacting within the social networks [23], [24]. Social capital is therefore largely seen as a characteristic of social groups rather than individuals and it is born of shared experience, which fosters mutual trust and reciprocity [25]. However, social capital is created in the connections among individuals in social groups, and it can therefore also be seen as an asset of individuals [26], [27]. Subsequently, the health effects of social capital may be observed both at the individual and collective levels [23], should be considered as both an individual and group attributes [11], and measured at both levels [28]. Social capital at the group (contextual) level has most often been measured by aggregating individual perceptions of social capital [23].

It has been argued that the workplace can be an important source of social capital [24], [29]. This argument has been supported by several studies finding that workplace social capital is associated with workers' health and health-related behaviors [12], [30], [31]. Workplace social capital may take on particular relevance in China. China is a familial and collectivistic society where the Chinese often utilize strong social networks composed of relatives, friends and acquaintances to obtain jobs [18]. RUMWs, in particular, were separated from their families and spend much time with co-workers in their place of work. Thus, w the workplace becomes a primary setting for cumulating social capital by RUMWs [32]. Consequently, for RUMWs, workplace social capital may be an important factor associated with better health outcomes. However, to our knowledge, there has been no published research examining the health effects of workplace social capital among RUMWs in China. Accordingly, the aim of the present study is to examine the association between workplace social capital (at individual- and workplace-levels) and HD among Chinese RUMWs. Based on the preceding literature review, our hypotheses are: (1) workplaces with higher levels of social capital will be associated with less HD among Chinese RUMWS, (2) individual level perceptions of workplace social capital, independent of collective perceptions of workplace social capital, will predict less HD.

## Methods

### 2.1 Population

The study was conducted in Shanghai, China during July 2012 to January 2013. Five thousand nine hundred and ninety-six RUMWs from 77 workplaces were randomly selected using a multi-stage sampling process. Firstly, seven districts (viz., Putuo, Pudong, Changning, Yangpu, Xuhui, Jiading and Qingpu) were randomly selected from the 17 districts that compose Shanghai. Four manufacturing companies, four hotel and catering industries, two construction worksites, and one entertainment company were selected in each selected district using a convenience-sampling method. Finally, rural-urban migrants who were aged 18 and older with a rural “Hukou”(that is, registered as a permanent rural resident), and currently working and living in Shanghai for at least 6 months [33] were selected to participate the current study. A self-administered questionnaire was distributed via the Human Resources department to all selected RUMWs, whom completed the questionnaire anonymously. The study was approved by the Institutional Review Board of the School of Public Health at Fudan University.

The total sample consisted of 5,996 subjects, representing 77 workplaces, who returned questionnaires. We excluded respondents with missing values on the social capital questions or items pertaining to drinking status, sex, or age, which resulted in an analytical sample of 5,318 subjects (88.7%). The average number of participants from each workplace (mean cluster size) was 69 (range: 35 to 251).

### 2.2 Measurements

#### 2.2.1 Problematic drinking

The Chinese version of Alcohol Use Disorders Identification Test (AUDIT) was used to assess HD [5]. The AUDIT consists of 10 items with score from 0 to 40. The AUDIT had high sensitivity and specificity and has been frequently used in workplace studies in China [3], [34]. Based on the guidelines provided in the AUDIT scoring manual [4] and previous studies [10], [34], a score of 8 or higher for men and 7 or higher for women is recommended an indicator for HD.

#### 2.2.2 Workplace social capital

Workplace social capital was assessed with a validated and psychometrically tested eight-item measure [30], [31], [35], Chinese version of Workplace Social Capital Scale. Based on the original scale [24], an initial translation into Chinese was done, followed by a translation back into English to verify the linguistic and semantic equivalence with the original scale. Prior psychometric evaluation in Chinese employees has demonstrated the scale to have high internal reliability (Cronbach's alpha 0.94) [35]. Using a 5-point Likert-scale, the participants assessed workplace social capital, defined as the shared values, attitudes, and norms of trust and reciprocity as well as practices of collective action in their workplace [24]. The Cronbach's alpha was 0.91 for the current sample. We assessed social capital in two alternative ways: (a) individual-level social capital, using each individual's own assessment, and to minimize subjectivity bias, (b) aggregated-level social capital, summing up the assessment of co-workers, but excluding the individual's own assessment. Both individual and aggregated-level social capital scores were divided into quartiles for the analysis, the highest quartile indicating the highest level of workplace social capital.

#### 2.2.3 Covariates

We selected the following variables as relevant confounders for statistical control: gender, age (10-year categories), marital status (married or cohabiting vs. other), current smoking (yes vs. no) and health insurance (have vs. have not). Salary was recorded in Yuan per month and categorized into five groups: <1500, 1500∼, 2500∼, 3500∼, 4500∼. Education attainment was categorized into elementary school, junior high school, senior high school and university or higher.

### 2.3 Statistic analyses

Our data had a multilevel structure comprised of RUMWs (at level 1) nested within workplaces (at level 2). We fitted the data using multilevel logistic regression models, adjusting for both individual- and workplace-level variables as fixed effects and allowing for heterogeneity between workplaces. Adjusted odds ratios (ORs) and their 95% confidence intervals (CIs) for HD were obtained for both the individual-level and aggregated-level scores of workplace social capital. The analysis proceeded according to the following steps [36]. After examining the workplace-level variance in HD without including any explanatory variables (empty model or null model), we examined the relationship between individual-level social capital and HD while adjusting for other individual-level covariates (model 1). Next, we included only aggregated-level social capital index and individual-level covariates (model 2). Then, we modeled individual- and aggregated-level social capital variables simultaneously (model 3). We used -2 log likelihood(-2LL) and Akaike information criterion(AIC) to compare the goodness-of-fit of each model [36]. The SAS version 9.1.3 program package was used for all analyses (SAS Institute, Inc., Cary, NC, USA). The multi-level analyses were performed using the GLIMMIX procedure.

## Results

### 3.1 Descriptive results

Demographic characteristics, the corresponding prevalence of HD, and univariate analyses are shown in Table 1. The overall prevalence of hazardous drinking was 10.6%, with males having a statistically higher prevalence of HD (18%) than females (2.2%). The prevalence was also higher among current smokers (26.2%) than among never/former smokers (5.3%), and higher among those with health insurance (11.3%) than among those without health insurance (8.5%). The prevalence was slightly, though statistically significantly, lower among those who were married/cohabiting (10.1%) than among their unmarried counterparts (12.4%). The rates of HD among RUMWs also differed by education level: those with the least education (elementary school) had the lowest rate (8.2%) whereas those with senior high school education had the highest rate of HD (13.1%). Hazardous drinking among RUMWs also significantly varied by salary level and by individual-level social capital social capital (both p<.05): the prevalence of HD ascended in conjunction with greater salary and declined in conjunction with greater individual perceptions of social capital.

**Table 1:** Demographic characteristics and hazardous drinking of the study subjects.

	*N*(%)	hazardous drinking *n*(%)	*p* value
**All**	**5318(100)**	**565(10.6)**	
**Sex**			
Men	2512(47.7)	502(18.3)	<.001
Women	2753(52.3)	54(2.2)	
**Age (year)**			
≤29	2132(40.1)	223(10.5)	.563
30–39	1377(25.9)	145(10.5)	
40–49	1365(25.7)	141(10.3)	
≥50	444(8.4)	56(12.6)	
**Education level**			
Elementary school	961(18.1)	79(8.2)	.002
Junior high school	2704(50.9)	276(10.2)	
Senior high school	1304(24.5)	171(13.1)	
University	349(6.6)	39(11.2)	
**Marital status**			
Married or cohabiting	3865(72.7)	390(10.1)	.039
Other	1453(27.3)	175(12.4)	
**Salary (Yuan/month)**			
∼1500	474(8.9)	35(7.4)	<.001
1500∼	2429(45.7)	177(7.3)	
2500∼	1667(31.4)	221(13.3)	
3500∼	415(7.8)	64(15.4)	
4500∼	333(6.3)	68(20.4)	
**Smoking status**			<.001
Never/former	3976(74.8)	208(5.3)	
Current	1342(25.2)	357(26.6)	
**Medical care insurance**			
Yes	4070(76.6)	459(11.3)	.006
No	1244(23.4)	106(8.5)	
**Individual-level social capital quartile**			
1^st^ (low)	1182(22.2)	145(12.3)	.032
2^nd^	1038(19.5)	122(11.8)	
3^rd^	1708(32.1)	173(10.1)	
4^th^ (high)	1390(26.1)	125(9.0)	

### 3.2 Multilevel analyses of the relationship between social capital and problematic drinking

Multilevel modeling results are shown in Table 2. The initial (empty) model indicated that there was statistically significant variation in the prevalence of HD across workplaces (χ^2^ = 182.42,p<.001). The intraclass correlation coefficients (ICC) was 0.223, indicating that 22.3% of variation in the prevalence of HD was explained by a random effect for workplaces.

**Table 2:** The odds ratios and 95% credible intervals for hazardous drinking associated individual-level and workplace-level social capital.

	Empty model	Model 1	Model 2	Model 3
	OR(95%CI)	OR(95%CI)	OR(95%CI)	OR(95%CI)
**Fixed effects**				
** Men (vs. Women)**		**5.26(3.77–7.35)**	**5.21(3.73–7.27)**	**5.21(3.74–7.28)**
** Age (year)**				
≤29		1	1	1
30–39		1.13(0.84–1.53)	1.12(0.83–1.51)	1.12(0.83–1.52)
40–49		1.06(0.77–1.47)	1.03(0.75–1.43)	1.05(0.76–1.45)
≥50		1.17(0.78–1.77)	1.16(0.77–1.74)	1.16(0.77–1.75)
** Education level**				
Elementary school		1.36(0.83–2.23)	1.36(0.83–2.24)	1.37(0.83–2.24)
Junior high school		1.28(0.84–1.95)	1.29(0.85–1.97)	1.29(0.84–1.96)
Senior high school		1.33(0.87–2.03)	1.36(0.89–2.08)	1.36(0.89–2.07)
University		1	1	1
** Married or cohabiting (vs. Other)**		0.76(0.57–1.02)	0.76(0.57–1.01)	0.76(0.57–1.01)
** Salary (Yuan/month)**				
∼1500		1	1	1
1500∼		0.79(0.52–1.20)	0.80(0.53–1.22)	0.79(0.52–1.20)
2500∼		1.07(0.70–1.63)	1.07(0.70–1.63)	1.06(0.70–1.61)
3500∼		1.19(0.73–1.95)	1.20(0.73–1.95)	1.19(0.73–1.94)
4500∼		**1.86(1.13–3.05)**	**1.86(1.13–3.04)**	**1.84(1.12–3.03)**
** Current smoking (vs. never/former)**		**3.29(2.67–4.06)**	**3.33(2.71–4.10)**	**3.30(2.68–4.07)**
** Medical care insurance (vs. no)**		1.27(0.99–1.65)	1.23(0.96–1.60)	1.26(0.98–1.63)
** Individual level social capital Quartile**				
4^th^ (high)		1		1
3^rd^		1.10(0.72–1.22)		**1.13(1.04–1.23)**
2^nd^		**1.15(1.02–1.53)**		**1.17(1.05–1.56)**
1^st^ (low)		**1.22(1.11–1.68)**		**1.26(1.13–1.72)**
** Workplace level social capital Quartile**				
4^th^ (high)			1	1
3^rd^			0.91(0.61–1.36)	0.83(0.55–1.25)
2^nd^			1.11(0.73–1.67)	1.09(0.72–1.64)
1^st^ (low)			1.31(0.90–1.92)	1.27(0.88–1.86)
**Random effects**				
Workplace-level variance (SE)	0.971(0.118)	0.444(0.100)	0.417(0.103)	0.411(0.103)
**Model fit**				
** -2LL**	3418.8	2932.1	2932.8	2927.3
** AIC**	3422.8	2970.1	2970.8	2968.3

*Note.* Statistically significant effects at *p*<.05 are shown in bold. -2LL: -2 Log Likelihood (smaller is better). AIC: Akaike information criterion (smaller is better).

The results of model 1 indicated that the adjusted odds of HD were greater among men (OR: 5.26, 95%CI: 3.77–7.35), workers in the highest salary category (OR: 1.86, 95%CI: 1.13–3.05) and current smokers (OR: 3.39, 95%CI: 2.67–4.06). Of focal interest, individual-level, perceived social capital was negatively associated with HD after controlling for all individual-level covariates. Compared to RUMWs in the highest quartile of perceived social capital, RUMWs in the lower three quartiles of perceived social capital exhibited progressively greater odds of problematic drinking, which were 1.10(95%CI: 0.92–1.22), 1.15(95%CI: 1.02–1.53), 1.22(95%CI: 1.11–1.68). However, it is possible that at least some of this effect could be due to between workplace variation in social capital contained within our measurement of individual-level perceptions of social capital. Hence, we estimated model 2 to examine whether aggregated-level social capital was associated with HD. There was no significant difference in the association of individual-level covariates and prevalence of HD between model 1 and model 2. Of focal interest, aggregated-level social capital was not significantly associated prevalence of HD. Compared with RUMWs in the fourth quartile (highest quartile) of aggregated-level social capital, the prevalence ratios for RUMWs in the third, second and first quartiles of aggregated-level social capital were 0.91 (95% CI: 0.61–1.36), 1.11 (95% CI: 0.73–1.67) and 1.31 (95% CI: 0.90–1.92) respectively (model 2).

In model 3, we added individual-level social capital to model 2. This quasi-contextual model allows us to assess whether individual perceptions of workplace social capital are associated with HD after controlling for workplace social capital, and also to assess whether there is a contextual effect of workplace-level social capital (i.e., a differential relationship between social capital and HD at the two levels). The results of this model indicate that the pattern of associations between individual-level covariates and prevalence of HD also didn't change meaningfully from models 1 and 2. After controlling for individual-level covariates, there was a positively graded association between individual-level social capital and odds of HD, but there remained no association between aggregated-level social capital and odds of HD. Compared with RUMWs in the fourth quartile (highest quartile) of individual-level social capital, the prevalence ratios for RUMWs in the third, second and first quartiles of individual-level social capital were 1.13 (95% CI: 1.04–1.23), 1.17 (95% CI: 1.05–1.56) and 1.26 (95% CI: 1.13–1.72) respectively.

## Discussion

To our best knowledge, this is the first multilevel modeling study that examines the association between social capital at work and HD among Chinese RUMWs. World Health Organization [1] estimated that the rates of alcohol use disorders in China were 6.9% and 0.2% among men and women, respectively. The current study found that the prevalence of HD were 18.3% and 2.2% among men and women, suggesting that immigrant status might be a risk factor to HD. Of focal interest, the findings suggest that individual-level social capital is significantly associated HD after controlling for demographic characteristics. By contrast, we did not find a contextual association between aggregated-level social capital and HD. Our findings are consistent with the findings in the UAS colleges; social capital exerts strong protective effects on alcohol abuse [37], [38]. However, Chuang et al. [39] found that social participation was positively associated with drinking among Taiwanese. The inconsistency of our findings with Chuang's study [39] may be because of the way drinking behavior and social capital were measured. Chuang et al. [39] measured drinking behavior by asking respondents whether they drink frequently and social participation by asking respondents to indicate their membership of clubs or associations. We used the Chinese version of Alcohol Use Disorders Identification Test (AUDIT) to assess HD and the validated Chinese version of eight-item measure [35] to assess workplace social capital, defined as the shared values, attitudes, and norms of trust and reciprocity as well as practices of collective action in their workplace [24]. The difference of research settings (community vs. workplace) may be another reason worthy of exploring in the future study.

There were several possible explanations why individual-level social capital was found to be associated with HD among RUMWs. First, drinking is often used as a coping mechanism to deal with stress [40], and RUMWs face high levels of stress from economic pressure, work load, and family separation [8]. Studies indicated high individual social capital at work could buffer the effects of stress by enhancing the individual's coping abilities [41], [42]. Furthermore, work-related relationships with migrant friends, employers and co-workers play an important role in the social capital of RUMWs [43] and may even be the primary source of their social capital. Secondly, social capital could increase the likelihood of accessing various forms of social support [11]. A previous study indicated that instrumental support and emotional support from co-workers were negatively associated with stress, smoking and drinking among Chinese workers [19]. Third, social capital has also been found to be associated with self-control (i.e. strong beliefs in the possibility to influence one's own health) [41], [44], [45]. Studies indicated that self-control was negatively associated with drinking [46]–[48]. Further research is needed to explore these and other mechanisms that might explain the association between workplace social capital and hazardous drinking found in this study.

The lack of association between workplace-level social capital and hazardous drinking may relate to social capital misclassification or measurement imprecision. Firstly, workplace-level social capital was aggregated by individual-level social capital of all co-workers in the same workplace. In some cases, informal work groups might provide a more accurate proxy for workplace-level social capital [41]. Thus, the assessment of all co-workers might be a less accurate reflection of social capital than an individual's own assessment. Secondly, Social capital in China resides largely in families or in other narrow circles of social relationships, which implies that people may only trust those who belong to the same in-group [49]. To RUMWs, migrant friends are their most important social networks in workplace [32], [43]. When individual-level social capital is aggregated up to the workplace level, its effect on hazardous drinking may tend to become diluted and less relevant. In this sense, a workplace climate characterized by the collective social capital accumulated by the mass of migrants in that workplace may be less important than an individual's personal experiences and perceptions.

Our study had several limitations that we should note. First, as is inherent in any cross-sectional study: no causal inferences can be drawn between workplace social capital and employee drinking behaviors. Second, workplace social capital may be affected by social capital outside workplaces, and vice versa. However, we didn't assess social capital from family members and relatives, which are main resources of social capital for RUMWs [43]. Indeed, a previous study has shown the importance of considering the social networks at work as well as outside companies on workers' health [50]. Thirdly, two limitations of the sampling methods should be noticed. We attempted to select varied types of workplaces in the second stage of sampling, but convenience-sampling method was used. Additionally, the sample of the current study was large, but some of eligible RUMWs in the selected workplaces may not respond to the survey in the third stage of sampling. These two limitations may limit the generalizability of the results to other industries not represented adequately in this study. Further longitudinal studies investigating the link between workplace social capital and problematic drinking among RUMWs from varied industries is warranted.

In conclusion, this study found a significant association between higher individual-level social capital and lower likelihood of problematic drinking among rural-urban migrant workers in China. By contrast, no clear association was found between workplace-level social capital and problematic drinking. As rural-urban migrant workers were separated from family members and have different experiences than urban residents, the workplace is an important context for building social capital. As workplace social capital is determined by workplace context and workers' socio-economic factors [51], a complex systems approach should be used [12]. The measures may involve (1) the implementation of various social activities or network interventions, such as peer support systems or social gatherings to increase network diversity or social participation; and (2) leadership development or collective mobilization efforts may be required to ensure employers provide equitable resources for social activities [52], [53]. Recognizing this, it is important that further longitudinal and intervention studies examine the possible link between workplace social capital and problematic drinking in Chinese workplaces.

## Supporting Information

S1 Table (XLS)

## References

[1] World Health Organization (2011) Global status report on alcohol and health. Geneva.

[2] Ma GZ, Hu DH, Luan XQ, Kong DC, Yang LH (2005) The Drinking Practice of People in China. Acta Nutrimenta Sinica 27:362–370.

[3] Li Q, Babor TF, Hao W, Chen X (2011) The Chinese translations of Alcohol Use Disorders Identification Test (AUDIT) in China: a systematic review. Alcohol Alcohol 46:416–423. 21467046 10.1093/alcalc/agr012 PMC3119458

[4] Babor TF, Biddle JH, Saunders JB, Monteir MG (2001) The Alcohol Use Disorders Identification Test: Guidelines for Use in Primary Care. Geneva: World Health Organization.

[5] Li B, Shen Y, Zhang B, zheng X, Wang X (2003) The Test of AUDIT in China. Chinese Mental Health Journal 17:1–3 (in Chinese)..

[6] Cui X, Rockett IR, Yang T, Cao R (2012) Work stress, life stress, and smoking among rural-urban migrant workers in China. BMC Public Health 12:979. 23151299 10.1186/1471-2458-12-979 PMC3584974

[7] China National Bureau Statistics (2001) Characteristics of Chinese Rural Migrants: 2000. Beijing: China National Bureau of Stattistics.

[8] Mou J, Griffiths SM, Fong H, Dawes MG (2013) Health of China's rural-urban migrants and their families: a review of literature from 2000 to 2012. Br Med Bull 106:19–43. 23690451 10.1093/bmb/ldt016

[9] Lin D, Su W, Deng L, Li X (2006) Alcohol intoxication and its contributing factors among young rural-to-urban migrants in Beijing. Psychological Development and Education 22:52–56.

[10] Zaller N, Huang W, He H, Dong YY, Song D, et al (2014) Risky Alcohol Use Among Migrant Women in Entertainment Venues in China. Alcohol Alcohol 49:321–326. 24452724 10.1093/alcalc/agt184 PMC3992910

[11] Kawachi I, Subramanian SV, Kim D (2008) Social Capital and Health. New York: Springer Science+Business Media.

[12] Kawachi I, Subramanian SV, Takao S (2013) Global Perspectives on Social Capital and Health. New York: Springer Science+Business Media.

[13] Harker Burnhams N, Parry C, Laubscher R, London L (2014) Prevalence and predictors of problematic alcohol use, risky sexual practices and other negative consequences associated with alcohol use among safety and security employees in the Western Cape, South Africa. Subst Abuse Treat Prev Policy 9:14. 24593946 10.1186/1747-597X-9-14 PMC3944609

[14] Marchand A (2008) Alcohol use and misuse: what are the contributions of occupation and work organization conditions? BMC Public Health 8:333. 18816388 10.1186/1471-2458-8-333 PMC2564939

[15] Mowbray O, Quinn A, Cranford JA (2014) Social networks and alcohol use disorders: findings from a nationally representative sample. Am J Drug Alcohol Abuse 40:181–186. 24405256 10.3109/00952990.2013.860984 PMC4004646

[16] Yip W, Subramanian SV, Mitchell AD, Lee DT, Wang J, et al (2007) Does social capital enhance health and well-being? Evidence from rural China. Soc Sci Med 64:35–49. 17029692 10.1016/j.socscimed.2006.08.027

[17] Wang H, Schlesinger M, Wang H, Hsiao WC (2009) The flip-side of social capital: the distinctive influences of trust and mistrust on health in rural China. Soc Sci Med 68:133–142. 18986744 10.1016/j.socscimed.2008.09.038

[18] Meng T, Chen H (2014) A multilevel analysis of social capital and self-rated health: Evidence from China. Health Place 27C:38–44. 10.1016/j.healthplace.2014.01.009 24531015

[19] Chen WQ, Wong TW, Yu IT (2008) Association of occupational stress and social support with health-related behaviors among chinese offshore oil workers. J Occup Health 50:262–269. 18408346 10.1539/joh.l7149

[20] Kawachi I, Kim D, Coutts A, Subramanian SV (2004) Commentary: Reconciling the three accounts of social capital. Int J Epidemiol 33:682–690 discussion 700–684. 15282222 10.1093/ije/dyh177

[21] Helliwell JF, Putnam RD (2004) The social context of well-being. Philos Trans R Soc Lond B Biol Sci 359:1435–1446. 15347534 10.1098/rstb.2004.1522 PMC1693420

[22] Coleman JS (1998) Foundations of social theory. MA, USA: Harvard University Press.

[23] Murayama H, Fujiwara Y, Kawachi I (2012) Social capital and health: a review of prospective multilevel studies. J Epidemiol 22:179–187. 22447212 10.2188/jea.JE20110128 PMC3798618

[24] Kouvonen A, Kivimaki M, Vahtera J, Oksanen T, Elovainio M, et al (2006) Psychometric evaluation of a short measure of social capital at work. BMC public health 6:251. 17038200 10.1186/1471-2458-6-251 PMC1618843

[25] Shortt SE (2004) Making sense of social capital, health and policy. Health Policy 70:11–22. 15312706 10.1016/j.healthpol.2004.01.007

[26] Coleman JS (1988) Social Capital in the Creation of Human Capital. American Journal of Sociology 94:S95–S120.

[27] Portes A (1998) Social Capital: Its Origins and Applications in Modern Sociology. Annual Review of Sociology 24:1–24.

[28] Szreter S, Woolcock M (2004) Health by association? Social capital, social theory, and the political economy of public health. Int J Epidemiol 33:650–667. 15282219 10.1093/ije/dyh013

[29] Kawachi I (1999) Social capital and community effects on population and individual health. Ann N Y Acad Sci 896:120–130. 10681893 10.1111/j.1749-6632.1999.tb08110.x

[30] Gao J, Nehl EJ, Fu H, Jia Y, Liu X, et al (2013) Workplace social capital and smoking among Chinese male employees: a multi-level, cross-sectional study. Prev Med 57:831–836. 24075818 10.1016/j.ypmed.2013.09.009

[31] Gao J, Weaver SR, Dai J, Jia Y, Liu X, et al (2014) Workplace social capital and mental health among Chinese employees: a multi-level, cross-sectional study. PLoS One 9:e85005. 24404199 10.1371/journal.pone.0085005 PMC3880334

[32] Chen X, Stanton B, Kaljee LM, Fang X, Xiong Q, et al (2011) Social Stigma, Social Capital Reconstruction and Rural Migrants in Urban China: A Population Health Perspective. Hum Organ 70:22–32. 21516266 10.17730/humo.70.1.k76047734m703500 PMC3080703

[33] Yang T, Xu X, Li M, Rockett IR, Zhu W, et al (2012) Mental health status and related characteristics of Chinese male rural-urban migrant workers. Community Ment Health J 48:342–351. 21394472 10.1007/s10597-011-9395-8

[34] Chen Y, Li X, Zhang C, Hong Y, Zhou Y, et al (2013) Alcohol use and sexual risks: use of the Alcohol Use Disorders Identification Test (AUDIT) among female sex workers in China. Health Care Women Int 34:122–138. 23311906 10.1080/07399332.2011.610535 PMC3563358

[35] Gao J, Jia Y, Wu X, Li G, Dai J, et al (2012) An exploratory study on relationship between social capital and health in workplace. Chinese Journal of Health Education 28:806–809 (in Chinese)..

[36] Wang J, Xie H, Jiang F (2008) Multilevel Models:Methods and Aplications. Beijing: Hiher Education Press (in Chinese).

[37] Weitzman ER, Chen YY (2005) Risk modifying effect of social capital on measures of heavy alcohol consumption, alcohol abuse, harms, and secondhand effects: national survey findings. J Epidemiol Community Health 59:303–309. 15767384 10.1136/jech.2004.024711 PMC1733052

[38] Weitzman ER, Kawachi I (2000) Giving means receiving: the protective effect of social capital on binge drinking on college campuses. Am J Public Health 90:1936–1939. 11111272 10.2105/ajph.90.12.1936 PMC1446447

[39] Chuang YC, Chuang KY (2008) Gender differences in relationships between social capital and individual smoking and drinking behavior in Taiwan. Soc Sci Med 67:1321–1330. 18667260 10.1016/j.socscimed.2008.06.033

[40] Dawson DA, Grant BF, Ruan WJ (2005) The association between stress and drinking: modifying effects of gender and vulnerability. Alcohol Alcohol 40:453–460. 15972275 10.1093/alcalc/agh176

[41] Kouvonen A, Oksanen T, Vahtera J, Vaananen A, De Vogli R, et al (2008) Work-place social capital and smoking cessation: the Finnish Public Sector Study. Addiction 103:1857–1865. 18705683 10.1111/j.1360-0443.2008.02315.x

[42] Sapp AL, Kawachi I, Sorensen G, LaMontagne AD, Subramanian SV (2010) Does workplace social capital buffer the effects of job stress? A cross-sectional, multilevel analysis of cigarette smoking among U.S. manufacturing workers. J Occup Environ Med 52:740–750. 20595910 10.1097/JOM.0b013e3181e80842 PMC2930819

[43] Li Y, Wu S (2010) Social networks and health among rural-urban migrants in China: a channel or a constraint? Health Promot Int 25:371–380. 20392708 10.1093/heapro/daq020

[44] Kouvonen A, Oksanen T, Vahtera J, Stafford M, Wilkinson R, et al (2008) Low workplace social capital as a predictor of depression: the Finnish Public Sector Study. Am J Epidemiol 167:1143–1151. 18413361 10.1093/aje/kwn067

[45] Pulkkinen L, Lyyra AL, Kokko K (2011) Is social capital a mediator between self-control and psychological and social functioning across 34 years? Int J Behav Dev 35:475–481.

[46] Piquero AR, Gibson CL, Tibbetts SG (2002) Does self-control account for the relationship between binge drinking and alcohol-related behaviours? Crim Behav Ment Health 12:135–154. 12459815 10.1002/cbm.492

[47] Foster DW, Young CM, Barnighausen TW (2014) Self-Control as a Moderator of the Relationship Between Drinking Identity and Alcohol Use. Subst Use Misuse 49:1340–1348. 24730565 10.3109/10826084.2014.901387 PMC4220739

[48] Botchkovar EV, Broidy L (2013) Parenting, self-control, and the gender gap in heavy drinking: the case of Russia. Int J Offender Ther Comp Criminol 57:357–376. 22859665 10.1177/0306624X11435318

[49] Allik J, Realo A (2004) Individualism-Collectivism and Social Capital. Journal of Cross-Cultural Psychology 35:29–49.

[50] Suzuki E, Takao S, Subramanian SV, Doi H, Kawachi I (2009) Work-based social networks and health status among Japanese employees. J Epidemiol Community Health 63:692–696. 19286690 10.1136/jech.2008.082453

[51] Oksanen T, Kawachi I, Kouvonen A, Takao S, Suzuki E, et al (2013) Workplace determinants of social capital: cross-sectional and longitudinal evidence from a Finnish cohort study. PLoS One 8:e65846. 23776555 10.1371/journal.pone.0065846 PMC3679109

[52] Better Together Work and Social Capital**.** John F. Kennedy School of Government, Harvard University. Available: http://bettertogether.org/pdfs/Work.pdf. Accessed 2014 Sep 21.

[53] Prusak L, Cohen D (2001) How to invest in social capital. Harv Bus Rev 79: 86–93147. 11408980

